# Preclinical Evaluation of a Novel PSMA-Targeted Agent ^68^Ga-NOTA-GC-PSMA for Prostate Cancer Imaging

**DOI:** 10.3390/tomography11030029

**Published:** 2025-03-07

**Authors:** Wenjin Li, Yihui Luo, Yuqi Hua, Qiaoling Shen, Liping Chen, Yu Xu, Haitian Fu, Chunjing Yu

**Affiliations:** 1 Department of Nuclear Medicine, Affiliated Hospital of Jiangnan University, No. 1000, Hefeng Road, Wuxi 214000, China; 6222809033@stu.jiangnan.edu.cn (W.L.); 622809044@stu.jiangnan.edu.cn (Y.L.); 7242808007@stu.jiangnan.edu.cn (Y.H.); 6212809031@stu.jiangnan.edu.cn (Q.S.); clpcyh007@126.com (L.C.); 6232818001@stu.jiangnan.edu.cn (Y.X.); 2 Wuxi School of Medicine, Jiangnan University, No. 1800, Lihu Avenue, Wuxi 214000, China

**Keywords:** prostate cancer, prostate-specific membrane antigen, PET imaging, ^68^Ga

## Abstract

**Objectives:** Prostate-specific membrane antigen (PSMA)-targeted radioligands are promising diagnostic tools for the targeted positron emission tomography (PET) imaging of prostate cancer (PCa). In present work, we aimed to develop a novel PSMA tracer to provide an additional option for prostate cancer diagnosis. **Methods:** Our team designed a new structure of the PSMA tracer and evaluated it with cellular experiments in vitro to preliminarily verify the targeting and specificity of ^68^Ga-NOTA-GC-PSMA. PET/CT imaging of PSMA-positive xenograft-bearing models in vivo to further validate the in vivo specificity and targeting of the radiotracer. Pathological tissue sections from prostate cancer patients were compared with pathological immunohistochemistry and pathological tissue staining results by radioautography experiments to assess the targeting-PSMA of ^68^Ga-NOTA-GC-PSMA on human prostate cancer pathological tissues. **Results:** The novel tracer showed high hydrophilicity and rapid clearance rate. Specific cell binding and micro-PET imaging experiments showed that ^68^Ga-NOTA-GC-PSMA displayed a high specific LNCaP tumor cell uptake (1.70% ± 0.13% at 120 min) and tumor-to-muscle (T/M) and tumor-to-kidney (T/K) ratio (13.87 ± 11.20 and 0.20 ± 0.08 at 60 min, respectively). **Conclusions:** The novel tracer ^68^Ga-NOTA-GC-PSMA is promising radionuclide imaging of PCa.

## 1. Introduction

Prostate cancer (PCa) is the second most frequent cancer and was the fifth leading cause of cancer death among men in 2020, with almost 1.4 million new cases and 375,000 deaths worldwide [1]. The 5-year relative survival rate for most people with local or regional PCa is nearly 100%. For people diagnosed with PCa that has spread to other parts of the body, the 5-year relative survival rate is 37% [2]. Therefore, early and accurate diagnosis of this disease is particularly important.

Molecular imaging is the visualization, characterization, and measurement of biological processes at the molecular and cellular levels in humans and other living systems [3,4,5]. Some of the key advantages of positron emission tomography (PET) in molecular imaging include high sensitivity, excellent temporal resolution, quantitative analysis, extended scan range specificity, dynamic imaging, multi-model imaging, and whole-body imaging. PET offers a variety of opportunities to address currently unsolved clinical issues [6]. Despite the inherent advantages of PET technology, SPECT also has some value for a wide range of applications. Worldwide, SPECT scanners far outnumber PET scanners, with the advantages of greater accessibility, lower cost, and availability of radiotracers that allow the study of a wider range of biological processes [7,8,9]. Radiolabeled drugs have been developed for the visualization of biological distribution in vivo, including uptake for tumor diagnosis, treatment, and efficacy evaluation. Prostate-specific membrane antigen (PSMA) is a highly expressed cell surface protein in PCa cells. Currently, clinical imaging agents, such as ^68^Ga-PSMA-11 [10,11] and ^68^Ga-PSMA-617 [12,13], are primarily composed of small-molecule radioligands utilized for positron emission tomography imaging. Several ^99m^Tc-PSMA radiopharmaceuticals have been in clinical trials, such as ^99m^Tc-PSMA-I&S, ^99m^Tc-HYNIC-iPSMA, and ^99m^Tc-MIP-1404, but they are not as effective as PSMA-PET tracers in terms of detection rate at low PSA levels and in small foci, as well as high uptake in the kidney, which makes image interpretation difficult [14,15,16]. Some studies have shown that ^99m^Tc-PSMA SPECT is prospective in bone metastasis and lymph node metastasis of prostate cancer [17,18,19]. ^99m^Tc-PSMA SPECT imaging agent developed as a simple, cost-effective imaging alternative to PSMA-PET [14,20,21]. These radioligands, which are structurally based on the Glu-urea-Lys (EuK) binding motif, have demonstrated a high sensitivity for the detection of primary tumors and metastatic lesions.

Developing a ^68^Ga-labeled PSMA-targeted tracer for PCa detection is currently an active research topic in nuclear medicine. ^68^Ga-PSMA-11 (Ki = 12.0 ± 2.8 nM [22]) and ^68^Ga-PSMA-617 (Ki = 2.34 ± 2.94 nM [23]) are mature PET/CT radioactive tracers, with the latter having better binding affinity, higher uptake in LNCaP bearing-tumors expressing PSMA, and faster clearance in background organs/tissues, including the kidneys, than the former. Hence, ^68^Ga-PSMA-617 was selected as the pharmacophore [23]. Our study designed a novel PSMA ligand NOTA-GC-PSMA, which is based on PSMA-617 and consists of three structural fragments: Glu-urea-Lys (EuK) targeting sequence, NOTA chelator, and a linker. The linker incorporated a functional group Cys (C)-Asp (D)-Lys (K), which is situated within the P1 structural region. Changes in the linker structure can affect the partial hydrophobic properties of the P1 structure, which is related to the strength of lipophilicity and affinity [24]. The inclusion of three hydrophilic amino acids in CDK might enhance the hydrophilicity of the ligand [23], thereby facilitating its excretion and reducing the potential for radioactive damage to non-target organs resulting from internal retention. NOTA-GC-PSMA could not only chelate with the radioactive metal ^68^Ga for PET imaging, but also has the potential to chelate with ^99m^Tc for SPECT imaging with N_3_S contained in its linkage portion of the moiety CDK, providing a viable option for different levels of medical care. In this study, we first evaluated the radiochemical and biological properties of the target agent radiolabeled with radioactive nuclide gallium-68 and tested it in cell and animal experiments. This study intended to provide a new probe for the clinical diagnosis of PCa.

## 2. Materials and Methods

### 2.1. Radioactive Synthesis and Purification of Probes

NOTA-GC-PSMA was designed by our team and synthesized by another company (Shanghai Apeptide Co., Ltd., Shanghai, China). The radionuclide ^68^Ga was eluted from a ^68^Ga/^68^Ge generator (Eckert & Ziegler, Berlin, Germany), with 0.1 M HCl as the fractionated eluent. The precursor NOTA-GC-PSMA (20 μg, 2 μg/μL) was mixed with the metallic cation ^68^GaCl_3_ (222–407 MBq, 1 mL) and sodium acetate buffer (0.15 M, 0.8–1.0 mL), with the pH of the reaction solution adjusted to 4.0–4.5. The reaction mixture was incubated at room temperature for 10 min, and the radiochemical purity was determined by reversed-phase HPLC (RP-HPLC).

### 2.2. In Vitro Stability Study

^68^Ga-NOTA-GC-PSMA (18.5 MBq, 0.5 mL) was mixed with phosphate-buffered saline (PBS, pH = 7.4, 0.5 mL) and fetal bovine serum (FBS 0.5 mL), respectively, to determine the stability of the tracer. The two solutions were maintained at 37 °C for different times (0, 0.5, 1, 2, and 4 h). FBS was precipitated with 0.5 mL of acetonitrile and removed by centrifugation (12,000 rpm, 5 min) after incubation. For stability analysis, 50 μL of the supernatants were subjected to radio-HPLC at each detection time point.

### 2.3. Determination of Lipid Water Partition Coefficient

^68^Ga-NOTA-GC-PSMA (0.4 MBq) was added to a centrifuge tube containing n-octanol (0.6 mL) and deionized water (0.6 mL). The mixture was shaken for 5 min and then centrifuged at 4000 rpm for 5 min. In brief, 0.5 mL of each upper and lower liquid layer (the upper layer was the n-octanol phase, and the lower layer was the water phase) was placed into an EP tube and subjected to γ-counter test radioactivity. Log*P* was calculated using the following formula: Log*P* = log10 [(counts in n-octanol phase/counts in water phase)].

### 2.4. Cell Lines with Culture Conditions

Two human prostate carcinoma cell lines, PSMA-positive LNCaP cells and PSMA-negative PC-3 cells, and cell culture reagents were obtained from the Procell Life Science & Technology Co., Ltd., Wuhan, China. In accordance with the instructions of the provider, the LNCaP cells were cultured in 1640 complete medium and the PC-3 PCa cells were cultured in DMEM complete medium with 10% FBS and 1% penicillin-streptomycin in 5% CO_2_ in an incubator at 37 °C. Subculture was performed with trypsin/EDTA solution (0.05%) when the culture reached 90% confluency.

### 2.5. Tumor Model

LNCaP tumor-bearing mice, modeled using B-NDG mice, were purchased from Biocytogen Pharmaceuticals Co., Ltd., Beijing China. All experiments for animal research were conducted in accordance with the principles laid out by the ethical committee of Jiangnan University (Animal Ethics: JN. No 20230830m0660315).

### 2.6. Analysis of the Affinity of NOTA-GC-PSMA to PSMA Through Surface Plasmon Resonance (SPR) Binding Assays

The SPR binding experiments were conducted in accordance with the biotin-labeling coupling method to test the affinity constant between PSMA protein (Human, PSA-H82Qb, ACRO Biosystems, Beijing, China) and small-molecule NOTA-GC-PSMA. The biotinylated PSMA protein was prepared at 5 μg/mL in HBS-N buffer and loaded onto an SA chip for conjugation under the conditions of 5 μL/min, 4200 s, and a coupling amount of approximately 6000 RU. Small-molecule NOTA was dissolved in HBS-EP buffer and diluted to the following concentrations: 12.5, 6.25, 3.125, 1.56, 0.79, and 0.38 μM. The running buffer for the affinity test was an HBS-EP buffer solution. The sequentially diluted solutions were loaded onto the SA chip conjugated with PSMA protein at 30 μL/min for 60 s and dissociation at 30 μL/min for 120 s, and the test curves were recorded. The signal values for 4 s before the end of the injection at each test concentration were read and fitted by Biacore T100 Evaluation Software 2.0.2 steady-state fitting to obtain the affinity constant.

### 2.7. Cellular Uptake and Blocking

For cellular uptake experiments, LNCaP and PC-3 PCa cells (3 × 10^5^ per well) were first seeded in six-well cell culture plates 48 h before experiments. After washing, ^68^Ga-NOTA-GC-PSMA (100 μL at 0.037 MBq) was added to the cell culture plates containing the fresh medium. The cells were then incubated for 30, 60, 90, and 120 min at 37 °C. The medium was removed, and the cells were washed with cold PBS (0.5 mL × 2) and lysed with RIPA lysis buffer (300 μL). Blocking experiments were performed by co-incubating the cells with ^68^Ga-NOTA-GC-PSMA in the presence of NOTA-GC-PSMA solution (3 µg/well). The radioactivity of the cells was measured by a γ-counter. The experiments were performed in triplicate.

### 2.8. Radiotoxicity

ICR male mice (n = 4/group) were injected with ^68^Ga-NOTA-GC-PSMA (5.55 MBq in 150 μL). For comparison, the control group mice were injected with 150 μL of normal saline. After injection, the mice were weighed, and their behavior, appearance, and physical signs were continuously observed for 14 days. After euthanasia, major organs were taken and fixed in 4% formalin. The organ specimens were trimmed and buried in paraffin wax. Tissue sections were prepared, stained with hematoxylin-eosin (H&E) staining, and observed under a light microscope.

At 14 days post-injection, the mice were weighed and euthanized and their main organs were harvested and fixed in 4% formalin. The organ samples were trimmed and embedded in paraffin. Histological sections were prepared, stained with H&E, and assessed under a light microscope.

### 2.9. Biodistribution and Pharmacokinetic Studies

ICR mice were injected with 200 μL of ^68^Ga-NOTA-GC-PSMA (3.70–5.55 MBq) via the tail vein. ^68^Ga-NOTA-GC-PSMA organ uptake was estimated by outlining the region of interest (ROI) on the image with PMOD software (version 4.3, PMOD Technologies, Zurich, Switzerland). The results were expressed as the percentage injected dose per gram (%ID/g, mean ± SD, n = 3).

The ^68^Ga-NOTA-GC-PSMA (3.7 MBq,100 μL) was administered intravenously via one of the lateral tail veins into ICR male mice (n = 6). A blood sample (5 μL) was collected via the orbital vein end at 5, 15, 30, 45, 60, 90, 120, 180, 240, and 300 min post-injection. The wet weight was immediately measured. The radioactivity of the blood samples was measured by a γ-counter, and the pharmacokinetics data were calculated using the MAS Studio for PK (Modelling and Simulation Studio for PK software 1.2.0).

### 2.10. PET Imaging

PET imaging was performed on a small-animal PET scanner (Ping Seng Healthcare Co., Ltd., Suzhou, China). Xenografted mice were injected with 4.0–5.0 MBq of ^68^Ga-NOTA-GC-PSMA via the tail vein. For the blocking group, the mice were pretreated with the precursor NOTA-GC-PSMA (100 μg/mice) 1 h in advance. All the mice were anesthetized with 1.5–2% isoflurane in a 0.5 L/min flow of oxygen. Dynamic images were collected for 0.5 h. Static imaging (10 min) was performed at 1 and 2 h after injection. The images were reconstructed using 3D ordered-subset expectation maximization and the point spread function with the attenuation correction of CT and subsequently processed by using PMOD (version 4.3, PMOD Technologies, Zurich, Switzerland). ROIs were drawn on the images of tumors and main organs, and corresponding signal levels were measured.

### 2.11. Autoradiography, H&E Staining, and Immunohistochemistry

The paraffin wax of the PCa pathological tissue was obtained from the pathological specimen bank of the Affiliated Hospital of Jiangnan University The tissue was sectioned into 4 μm-thick slices. The sections were deparaffinized in xylene and rehydrated in a graded ethanol series.

An immunohistochemistry (IHC) assay was conducted on the prostate tumor tissues following a previous protocol to determine PSMA expression. Antigen retrieval was performed with a steam cooker using a retrieval buffer (Tris-EDTA, BL617A) for 20 min at 100 °C. The tissues were treated with 3% H_2_O_2_ for 10 min to block endogenous peroxidase, followed by incubation with 5% BSA for 1 h at room temperature to block nonspecific binding. The tissue slices were then incubated with primary anti-PSMA rabbit monoclonal antibody (anti-PSMA EPR6253, Abcam, ab133579; dilution for 1:300 in volume) overnight at 4 °C. After washing, the tissues were incubated with the secondary antibody (GK5007, Gene Technology (Shanghai) Co., Ltd., Shanghai, China) for 15 min at room temperature and washed with PBS three times. Finally, the slices were mounted on glass slides with neutral balsam and visualized under an inverted microscope (Olympus; ×100) at different view fields.

Consecutive sections were subjected to autoradiography and H&E staining for the morphological characterization of tissue pathology to determine radiotracer distribution. The paraffin sections from different tissues were stained with H&E staining in accordance with standard procedure.

For in vitro PSMA autoradiography using ^68^Ga-NOTA-GC-PSMA, the sections were incubated with 16 μCi/mL ^68^Ga-NOTA-GC-PSMA at room temperature in Tris-HCl buffer (170 mmol/L; pH 8.2) containing 1% BSA to inhibit endogenous proteases. The incubated sections were washed twice for 5 min in cold Tris-HCl (170 mmol/L; pH 8.2) containing 0.1% BSA, then completely immersed for 10 s in Tris-HCl (170 mmol/L; pH 8.2), and quickly dried. The radioactivity bound to the sections was evaluated using the storage phosphor system (Cyclone Plus, PerkinElmer, Waltham, MA, USA).

### 2.12. Graphical and Statistical Analysis

All graphs and statistical analyses were generated using GraphPad Prism 8.0, Origin 2021, or SPSS 26. All quantitative data were expressed as the mean ± the standard deviation (mean ± SD). Statistical significance was analyzed using the independent *t*-test. Differences with a *p*-value less than 0.05 were considered statistically significant.

## 3. Results

### 3.1. Radioactive Labeling and Quality Control of Probe

**Figure 1:**
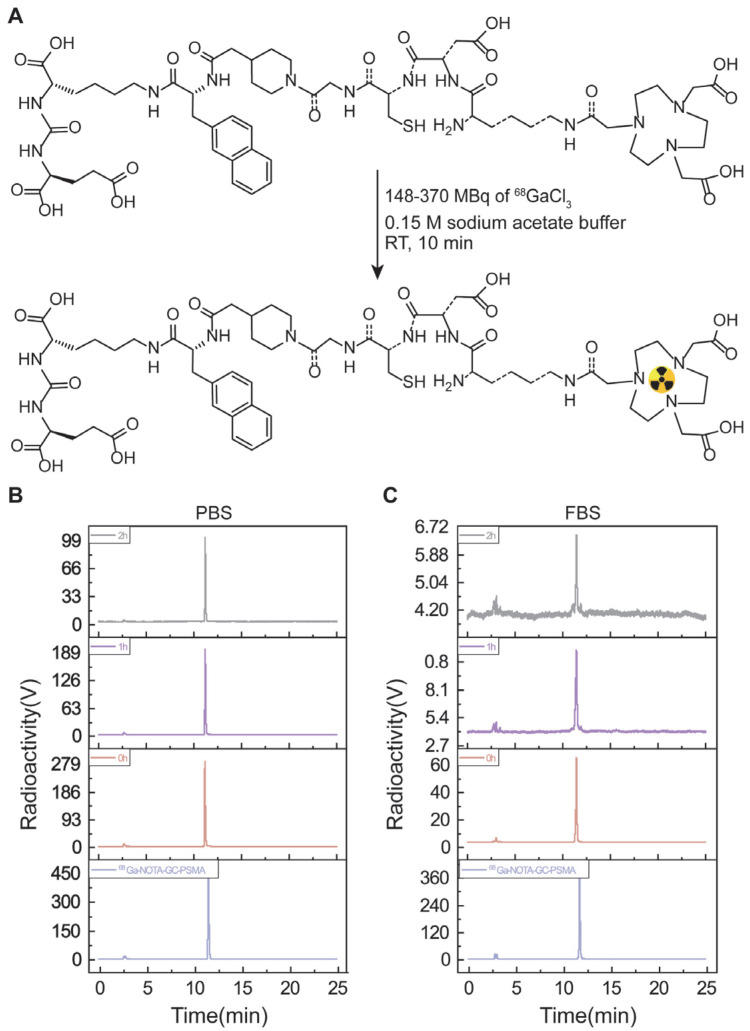
The labeling process and stability determination of ^68^Ga-NOTA-GC-PSMA. (**A**) ^68^Ga-NOTA-GC-PSMA. Stability of the ^68^Ga-NOTA-GC-PSMA in PBS (**B**) and FBS (**C**) with radio-HPLC.

This study designed a novel structural PSMA ligand named NOTA-GC-PSMA precursor (Figure 1A). Its molecular weight was approximately 1330.48 (Figure S1). NOTA-GC-PSMA was reacted with ^68^GaCl_3_ at room temperature for 10 min to obtain ^68^Ga-NOTA-GC-PSMA, which was produced with a radiochemical yield of more than 94.2% ± 1.9%, a radiochemical purity of more than 96.3% ± 3.37%, and the molar activity of 17.24 ± 4.96 GBq/µmol. The in vitro stability of ^68^Ga-NOTA-GC-PSMA in PBS and FBS was confirmed by a radiochemical purity of more than 96% over 2 h at 37 °C (Figure 1B,C). The Log*P* value of probe ^68^Ga-NOTA-GC-PSMA was −2.598 ± 0.16 (n = 3), indicating that the probe was hydrophilic. The affinity of NOTA-GC-PSMA was calculated as KD = 0.51 μM (Figure S3).

### 3.2. Cellular Uptake and Blocking

**Figure 2:**
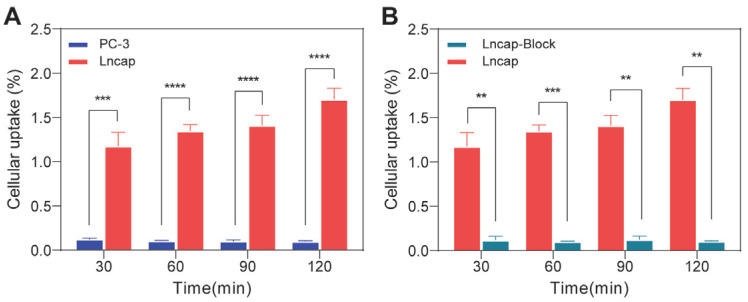
Cellular uptake and blocking assays. (**A**) LNCaP cells and PC-3 cells were incubated at ^68^Ga-NOTA-GC-PSMA for 30, 60, 90, and 120 min. (**B**) The LNCaP cell-blocking group was given an overdose of NOTA-GC-PSMA for 1 h pre-incubated and then incubated at ^68^Ga-NOTA-GC-PSMA for 30, 60, 90, and 120 min, and then measured by a γ-counter. The cellular uptake (%10^5^ cells) is the percentage of cellular radioactivity in total input radioactivity. All data are means ± SD (** *p* < 0.005, *** *p* < 0.0005, **** *p* < 0.00005, n = 3).

A cellular uptake study was performed to evaluate the binding efficiency of ^68^Ga-NOTA-GC-PSMA toward LNCaP cells and PC3 cells in vitro and to initially validate the ability of ^68^Ga-NOTA-GC-PSMA for targeting-PSMA. As shown in Figure 2A, Compared with PC-3 cells, ^68^Ga-NOTA-GC-PSMA exhibited significant radioactivity accumulation in LNCaP cells and displayed a gradually increasing trend at 30, 60, 90, and 120 min (*p* < 0.0005). In vitro cellular experiments showed that the probe had high uptake in LNCaP cells (1.70% ± 0.13% at 120 min) and almost no uptake in PC-3 cells (0.10% ± 0.01% at 120 min), thereby validating that ^68^Ga-NOTA-GC-PSMA could effectively target PSMA. This finding facilitated the design of subsequent in vivo experiments to substantiate the probe’s efficacy. After pretreatment with 3 μg excess precursor NOTA-GC-PSMA, ^68^Ga-NOTA-GC-PSMA specific binding was blocked in the LNCaP cells (0.11% ± 0.004%, 120 min). The blocking result further confirmed the specificity of the tracer binding to PSMA-positive cells (Figure 2B).

### 3.3. In Vivo Radiotoxicity Study

**Figure 3:**
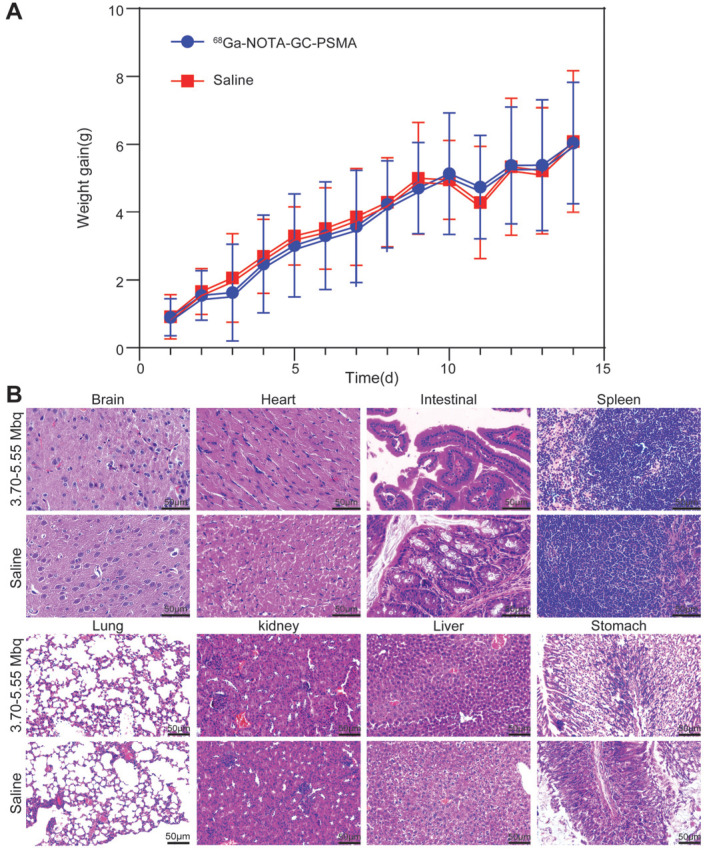
Radiotoxicity evaluation of ^68^Ga-NOTA-GC-PSMA (3.70–5.5 MBq/mouse) in ICR mice (n = 4/group). (**A**) Experimental and control groups were injected with ^68^Ga-NOTA-GC-PSMA and saline, respectively, and the body weight changes were observed for 14 consecutive days. (**B**) H&E staining results of major organs (brain, heart, liver, spleen, lungs, kidneys, small intestine, stomach) of mice in the ^68^Ga-NOTA-GC-PSMA and saline groups.

The mice injected with either ^68^Ga-NOTA-GC-PSMA or normal saline did not die within 14 days. No significant difference in body weight, diet, excretion, activity, mental state, or skin condition was found between the two groups during the observation period. The trend of body weight change was the same in the two groups (Figure 3A). No significant difference in the H&E staining results for the main organs, such as the brain, heart, liver, spleen, lungs, kidneys, small intestine, and stomach tissues, was observed between the two groups (Figure 3B). These results indicated that ^68^Ga-NOTA-GC-PSMA has a good safety profile, warranting further investigation.

### 3.4. In Vivo Pharmacokinetics and Biodistribution Studies

**Figure 4:**
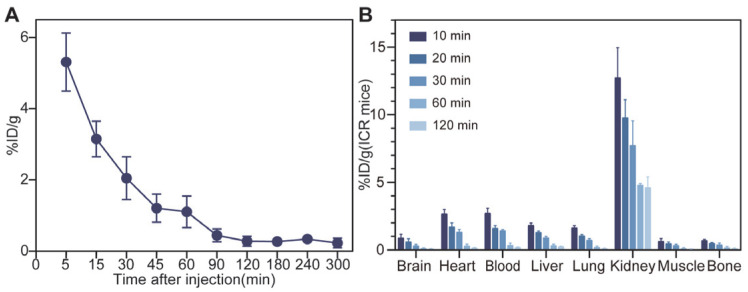
(**A**) The blood half-life of ^68^Ga-NOTA-GC-PSMA was fitted to 39 min by measuring the radioactivity of the mouse blood samples at different time points (%ID/g, mean ± SD, n = 6) using the MAS Studio for PK software. (**B**) ICR mice were injected with ^68^Ga-NOTA-GC-PSMA for micro-PET/CT imaging, and ROIs were sketched on the images by PMOD software to estimate the major organ and tissue biodistribution of ^68^Ga-NOTA-GC-PSMA (%ID/g, mean ± SD, n = 3).

The pharmacokinetic parameters of ^68^Ga-NOTA-GC-PSMA radiotracers in normal ICR mice were determined to investigate their blood clearance (Figure 4A). The radioactivity in the mouse blood indicated that the radiotracers were eliminated rapidly in vivo. The plasma half-life time (t_1/2_) of ^68^Ga-NOTA-GC-PSMA was 39 min, and the biodistribution in normal male ICR mice was summarized in Figure 4B (Table S5). In normal ICR mice, the accumulation of ^68^Ga-NOTA-GC-PSMA was high in the kidneys from 12.73 ± 2.22 %ID/g at 10 min to 4.63 ± 0.78 %ID/g at 120 min post-injection (Figure 4B), indicating that ^68^Ga-NOTA-GC-PSMA may be cleared from the body through renal metabolism (Figure 4B). In addition, the radioactivity was eliminated quickly, and a relatively low distribution of radioactivity was observed in other tissues at 120 min after injection (<0.25 %ID/g).

### 3.5. PET Imaging

**Figure 5:**
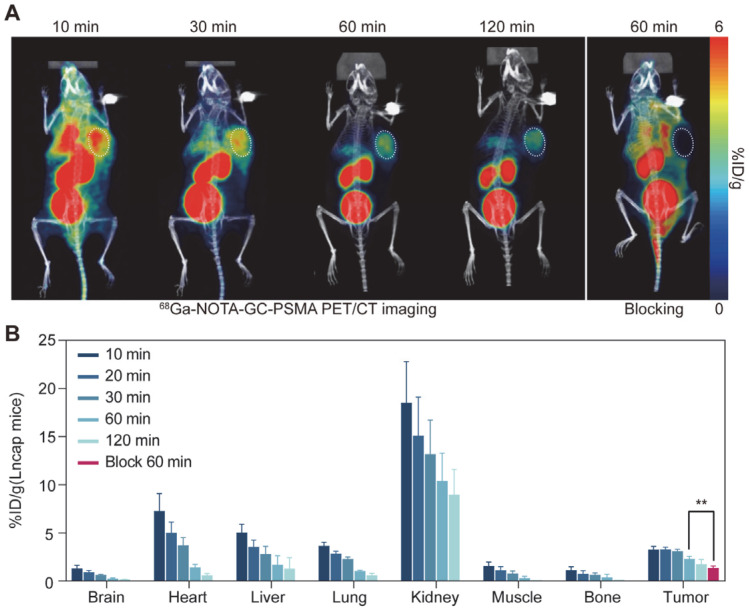
LNCaP tumor-bearing mice (n = 3/group) were injected with ^68^Ga-NOTA-GC-PSMA for micro-PET/CT imaging. (**A**) LNCaP tumor-bearing mice were injected with ^68^Ga-NOTA-GC-PSMA (3.0~5.5 MBq/mouse) and underwent micro-PET/CT imaging, and the blocking group was pre-injected with excess NOTA-GC-PSMA. (**B**) PET/CT images of LNCaP mice were outlined by PMOD software to quantify ^68^Ga-NOTA-GC-PSMA biodistribution in major organs and tissues. (** *p* < 0.005).

Consistent with the results of the in vitro cellular experiments, the results of the in vivo animal experiments showed that the PSMA-expressing positive LNCaP tumor model showed significant uptake of ^68^Ga-NOTA-GC-PSMA. ^68^Ga-NOTA-GC-PSMA PET/CT imaging showed a significant concentration of radioactivity in the LNCaP tumors (Figure 5A). Rapid and high tumor accumulation of ^68^Ga-NOTA-GC-PSMA was observed at 3.01 ± 0.56 %ID/g in LNCaP tumors as early as 10 min and remained relatively stable within approximately 120 min. The uptake was the highest in the kidneys, decreasing within 120min, from 16.69 ± 3.96 %ID/g (at 10 min) to 8.95 ± 2.63 %ID/g (at 120 min) in LNCaP tumor-bearing mice (Table S6). The tumor-to-muscle (T/M) and tumor-to-kidney (T/K) ratios were 13.87 ± 11.20 %ID/g and 0.20 ± 0.08 %ID/g at 60 min, respectively (Table S6). For common ^68^Ga-labelled compounds, international guidelines and studies recommend an uptake time of 60 min for PET imaging [25]. For ^68^Ga-NOTA-GC-PSMA, LNCaP tumor-bearing mice showed high T/M and T/K at 60 min, as well as high radioactivity accumulation at the tumor site. PET scans show the best imaging results (Figure 5A). Based on the above factors, the images were optimal at 60 min after injection. After blocking with NOTA-GC-PSMA, the uptake of ^68^Ga-NOTA-GC-PSMA in the LNCaP tumor model was reduced (1.05 ± 0.58 %ID/g at 60 min after blocking, *p* < 0.005) (Figure 5B), indicating that ^68^Ga-NOTA-GC-PSMA can specifically target PSMA-positive tumors in vivo.

### 3.6. Autoradiography

**Figure 6:**
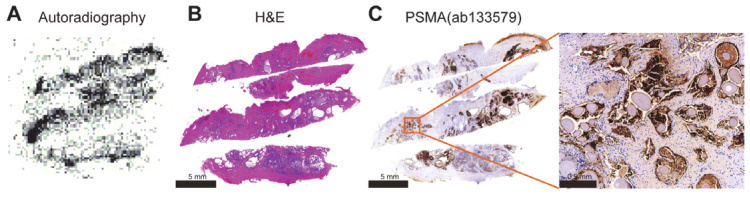
Association of ^68^Ga–NOTA-GC-PSMA with PSMA expression at the tumor tissue level. Autoradiography (**A**), H&E staining (**B**), and PSMA immunohistochemical staining (**C**) of the tumor tissue from a patient with PCa.

Adjacent slices were subjected to autoradiography (Figure 6A) with ^68^Ga-NOTA-GC-PSMA and subsequent staining (Figure 6B,C) to further investigate the correlation between the radioactivity distribution and PSMA expression (Figure 6C). As expected, the results showed the clustering of ^68^Ga-NOTA-GC-PSMA signals in the pathological sites with high PSMA expression, further confirming the targeting and specificity of this probe.

## 4. Discussion

The survival time of patients with PCa is closely related to the staging of malignant tumors during clinical diagnosis. Therefore, early detection, diagnosis, accurate evaluation, and standardized treatment are important to improve the prognosis of these patients. The traditional examinations used for clinical evaluation and staging include X-ray, CT, MRI, and bone scan, all of which rely on morphological standards. In addition, these methods are limited in their early diagnostic specificity and assessment of the true extent of cancer [26,27]. PET/CT has recently gained a significant role in the diagnostic imaging of PCa.

PSMA is an established PCa marker, and its expression in PCa tissues increases by 100–1000 times compared with that in normal tissues. Thus, PSMA has been considered a promising biological target for imaging diagnostics and targeted radionuclide therapy for PCa and its metastases. PSMA PET is now part of the diagnostic flowchart for PCa in international guidelines [6,28]. Several PSMA radioactive tracers are currently available, and many are under research, increasing the global availability of PSMA-PET imaging [29].

In this study, NOTA-GC-PSMA was developed based on the glutamate-urea structure, which exhibits a strong PSMA-binding affinity for PSMA in the nanomolar range and allows for various structural modifications within the non-pharmacophore pocket [30]. In particular, EuK, which is the most common affinity group for the development of PSMA-targeted probes [30,31,32,33], has been widely studied for the structural modification of PSMA-targeted inhibitors, such as PSMA-11 [34] and PSMA-617 [23]. Most structural modifications of the linker have been shown to be more acceptable than alterations targeting the active site. Linker modification is an attractive area when looking for more effective PSMA inhibitors and improving pharmacokinetics [34] and is regarded as an extension of the original EuK pharmacophore. Studies have shown that the length, polarity, size, flexibility, presence of aromatic and hydrophobic functional groups, and distance between the active sites play an important role in changing the affinity, internalization rate, and imaging contrast [35]. The arrangement order and spatial configuration of CDK might provide flexibility and adaptability to the connection points, help achieve an effective connection between pharmacophore and NOTA, and maintain the appropriate configuration of the whole molecule. The sulfur-containing group of cysteine can form disulfide bonds, which help to enhance the stability of the linking site and prevent the fracture or dissociation of the linking structure. The linking units of cysteine, aspartic acid, and lysine are polar amino acids with hydrophilic properties, which can affect the overall hydrophilicity. ^68^Ga-NOTA-GC-PSMA has good hydrophilicity (Log*P* −2.598 ± 0.16), which contributes to the rapid plasma clearance of radioactive tracers in vivo and reduces radiation damage to nontarget organs and tissues. The cell experiment confirmed the specificity of NOTA-GC-PSMA. The uptake was the highest in LNCaP cells and can be inhibited by NOTA-GC-PSMA (*p* < 0.05). The plasma half-life of ^68^Ga-NOTA-GC-PSMA (Figure 4A t_1/2_ 39 min) is similar to that of ^68^Ga-PSMA-11 (t_1/2_ 30.24 min [36]), indicating that the former is a tracer suitable for imaging diagnosis. In the mouse toxicity experiment, no adverse events or pathological changes related to acute radiation toxicity were observed, indicating that ^68^Ga-NOTA-GC-PSMA has good safety. However, NOTA-GC-PSMA showed moderate binding affinity. In general, the mechanistic explanation for the difference in the inhibition constants of PSMA inhibitors underscores the importance of the P1 region structure. Differences in the linking or distal groups result in varying inhibitory effectiveness. The P1 region structure partially contributes to physicochemical and pharmacokinetic characterization by interactions with non-pharmacodynamic sites of proteins [37]. Hydrophobic distal functionalities and relatively short linkers engage nonpolar residues in the entrance funnel, leading to high affinities [35]. Research revealed that the lipophilicity of the linker unit was associated with improved binding properties [35,38,39]. The linker of ^68^Ga-NOTA-GC-PSMA is long, and the newly added connection units Asp, Cys, and Lys are hydrophilic amino acids that will affect the affinity of the probe.

It was preliminarily verified by in vitro cellular experiments that ^68^Ga-NOTA-GC-PSMA could specifically target PSMA. We further explored the in vivo biodistribution of the proposed radioactive tracers and their applicability to micro-PET imaging in LNCaP tumor-bearing mice. Compared to the conventional radiotracer ^18^F-FDG, ^68^Ga-NOTA-GC-PSMA was PSMA-targeted and showed non-inferior uptake to ^18^F-FDG in the LNCaP tumor model (3.1 ± 0.6 %ID/g [40]) at the LNCaP tumor site. Owing to the moderate affinity of NOTA-GC-PSMA, the tumor site of LNCaP tumor-bearing mice showed specific uptake of ^68^Ga-NOTA-GC-PSMA (3.10 ± 0.20 %ID/g at 30 min and 2.28 ± 0.27 %ID/g at 60 min). An important factor may be that most injected tracer molecules are excreted by the kidneys before they can bind to the tumor. Given that our tracer can be excreted quickly, its tumor absorption is reduced [41]. In addition, the heart and lungs have high uptake in the early stage but exhibit a rapid decline in the late stage due to the high hydrophilicity and rapid blood clearance of ^68^Ga-NOTA-GC-PSMA. The nonspecific uptake by the liver may be related to the hepatobiliary excretion of ^68^Ga-NOTA-GC-PSMA and the negative charge of the linker. The overall lipophilicity of affinity molecules, the local distribution of lipophilic and hydrophilic amino acids, and the presence of positively charged N-terminal chelating agents may all be related to elevated liver uptake [35,39]. This finding was also verified in our experiment: ^68^Ga-NOTA-GC-PSMA has lower hydrophilicity than ^68^Ga-PSMA-11 (−4.51 ± 0.08) and ^68^Ga-PSMA-617 (−3.16 ± 0.14) [42], and the liver uptake of ^68^Ga-NOTA-GC-PSMA (1.69 ± 0.91 %ID/g) in LNCaP tumor-bearing mice at 60 min after injection was higher than that of ^68^Ga-PSMA-11 (0.56 ± 0.35 %ID/g) and ^68^Ga-PSMA-617 (0.63 ± 0.16 %ID/g) [42]. Despite its moderate binding affinity, the specific uptake of NOTA-GC-PSMA was observed in the PSMA-expressing LNCaP tumor xenografts with good tumor-to-background contrast. After injection, the micro-PET/CT results are basically consistent with the biological distribution, ^68^Ga-NOTA-GC-PSMA rapidly accumulated and metabolized in LNCaP tumors, and PSMA expression and the excretion of radioactive tracers caused the relatively high uptake in the kidneys. Compared with the PSMA-targeted PET tracers ^68^Ga-PSMA-11 (204 ± 70.6 %ID/g at 60 min) and ^68^Ga-PSMA-617 (29.2 ± 5.14 %ID/g at 60 min) [42], ^68^Ga-NOTA-GC-PSMA showed lower renal uptake (only 10.38 ± 2.90 %ID/g at 60 min) and higher T/K and T/M ratios. Hence, the proposed tracer might improve the sensitivity in detecting metastatic PCa lesions between kidneys. Owing to the relatively high specific uptake of ^68^Ga-NOTA-GC-PSMA in tumors and its rapid clearance from the blood and kidneys, the T/K and T/M ratios of ^68^Ga-NOTA-GC-PSMA were higher than that of ^68^Ga-PSMA-11 (T/K 0.22 ± 0.08 vs. 0.09 ± 0.04 at 30 min, 0.20 ± 0.08 vs. 0.04 ± 0.02 at 60 min; T/M 5.10 ± 2.27 vs. 4.17 ± 1.91 at 30 min, 13.87 ± 11.12 vs. 4.67 ± 2.38 at 60 min) [43], which made tumors easy to detect and was potentially helpful in detecting tumors closer to the kidneys. In addition, rapid clearance reduces the toxicity of radioactive drugs in the blood and kidneys. The high PSMA expression in the patient PCa sections, as indicated by immunohistochemistry (Figure 6C), was consistent with the high accumulation area in ^68^Ga-NOTA-GC-PSMA tumors, as revealed by autoradiography (Figure 6A). This finding confirmed the tumor targeting and specificity of ^68^Ga-NOTA-GC-PSMA in human histology. On the basis of the above results, this radioactive tracer may have the potential for further development and improvement. This study provides preliminary data support for subsequent research to further optimize and improve the trace’s affinity to increase tumor-specific uptake and reduce the nonspecific uptake of other organs.

This study has some limitations. No experiments were designed to verify the effect of the precursor junction structure on overall lipophilicity and affinity. Animal models of the negative control group were not designed in animal experiments. Only the cell line-derived xenograft (CDX) model was used; future research could employ the patient-derived tumor xenograft (PDX) model for scientific and conclusive outcomes. The N_3_S formed by the CDK structure added to the connection part can be chelated with ^99m^Tc [15,44] for SPECT imaging, which has advantages such as greater accessibility, low cost, and radiometric tracers for studying a wide range of biological processes [7,8,9]. Hence, SPECT is considered a practical routine test in nuclear medicine. ^99m^Tc has become an important radionuclide in clinical SPECT imaging [7,8,9,45]. In conjunction with the current data, our study will continue to optimize NOTA-GC-PSMA and refine preclinical studies related to ^99m^Tc-NOTA-GC-PSMA.

## 5. Conclusions

Our team designed a novel ^68^Ga-labeled radiotracer for the non-invasive detection of PCa. As a novel ^68^Ga-labeled PSMA inhibitor, the radiotracer can be produced by convenient manual operation in good radiolabeling yield and has no radio-related acute toxicity in vivo. On the basis of these promising results, including the rapid in vivo clearance and high T/K and T/M ratios in mouse models, ^68^Ga-NOTA-GC-PSMA shows good imaging performance for PCa. Its feasibility must be further evaluated.

## 6. Associated Content

### Supporting Information

The chemical structure of NOTA-GC-PSMA, LC-MS, and HPLC, the chromatogram of NOTA-GC-PSMA, the radio-HPLC chromatogram of ^68^Ga-NOTA-GC-PSMA, the analysis method of HPLC, and the figure of binding between NOTA-GC-PSMA and PSMA was tested through surface plasmon resonance. Cellular experimental data of ^68^Ga-NOTA-GC-PSMA, the pharmacokinetic data of ^68^Ga-NOTA-GC-PSMA, the blood sample data of the ^68^Ga-NOTA-GC-PSMA in ICR mice, PET/CT imaging and tissue biodistribution data of ^68^Ga-NOTA-GC-PSMA in ICR mice, and tissue biodistribution data of ^68^Ga-NOTA-GC-PSMA in LNCaP tumor-bearing mice (PDF).

## Data Availability

The data presented in this study are available on request from the corresponding author due to privacy.

## Supplementary Materials

The following supporting information can be downloaded at: https://www.mdpi.com/article/10.3390/tomography11030029, Figure S1: The structure and LC-MS of NOTA-GC-PSMA; Figure S2: HPLC of NOTA-GC-PSMA and ^68^Ga-NOTA-GC-PSMA; Figure S3: The binding between NOTA-GC-PSMA and PSMA was tested through surface plasmon resonance; Figure S4: PET/CT imaging of ICR mice (n = 3) with injecting ^68^Ga-NOTA-GC-PSMA; Table S1: HPLC analysis method of NOTA-GC-PSMA and ^68^Ga-NOTA-GC-PSMA; Table S2: Cellular experimental data of ^68^Ga-NOTA-GC-PSMA; Table S3: The pharmacokinetic data of ^68^Ga-NOTA-GC-PSMA was tested by MAS; Table S4: The blood sample data of the ^68^Ga-NOTA-GC-PSMA in ICR mice (n = 6); Table S5: Biodistribution of ^68^Ga-NOTA-GC-PSMA in ICR mice (%ID/g, Mean ± SD, n = 5); Table S6: The uptake of ^68^Ga-NOTA-GC-PSMA in major organs of LNCaP tumor-bearing mice (%ID/g, Mean ± SD, n = 5).
