# Supplementary material for: Preclinical Evaluation of a Novel PSMA-Targeted Agent 68Ga-NOTA-GC-PSMA for Prostate Cancer Imaging
Source: Tomography. 2025 Mar 7;11(3):29. doi: 10.3390/tomography11030029 (PMC11946674; doi:10.3390/tomography11030029)
Supplement: Supplementary file 1 [file tomography-11-00029-s001.zip › supporting information.docx]

Supporting information


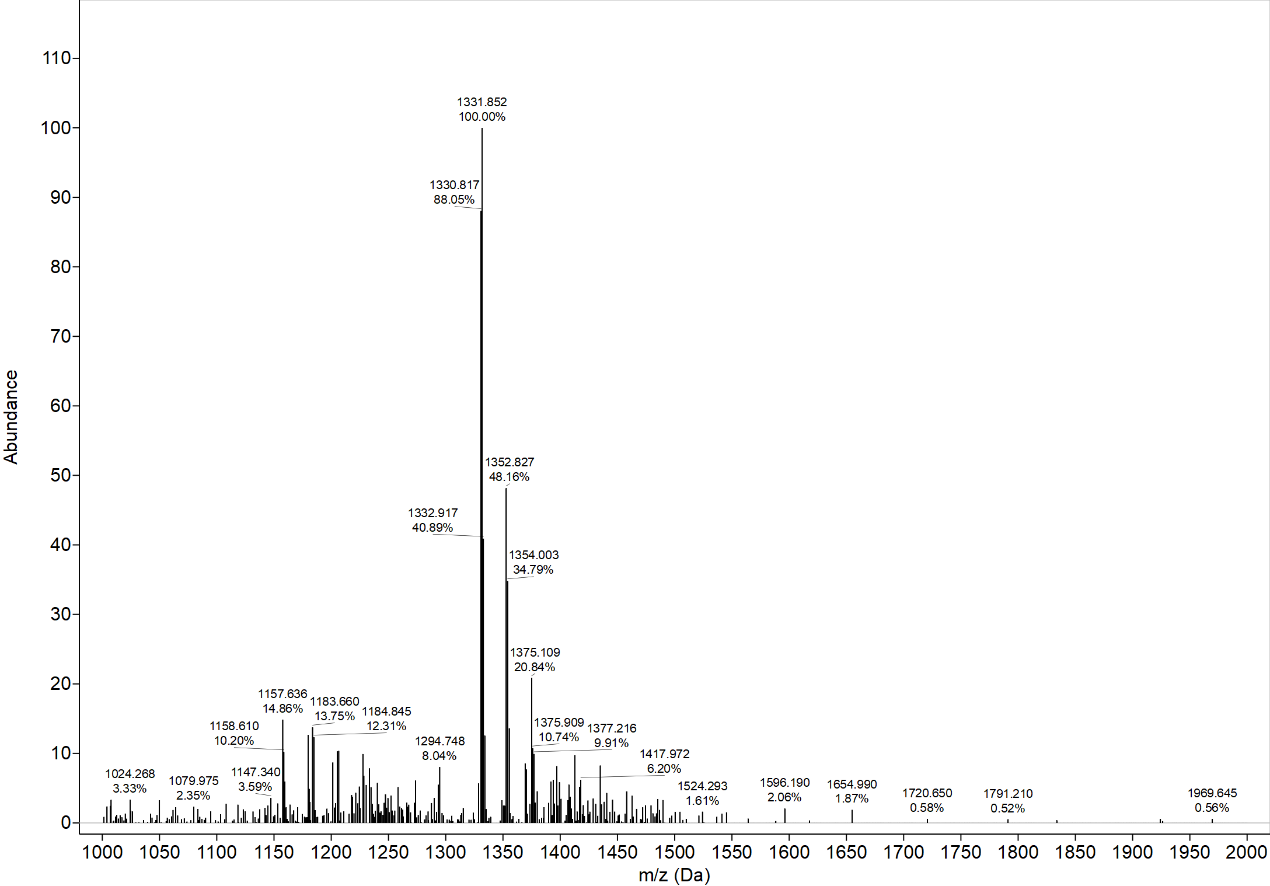

Figure S1. The structure and LC-MS of NOTA-GC-PSMA.

**
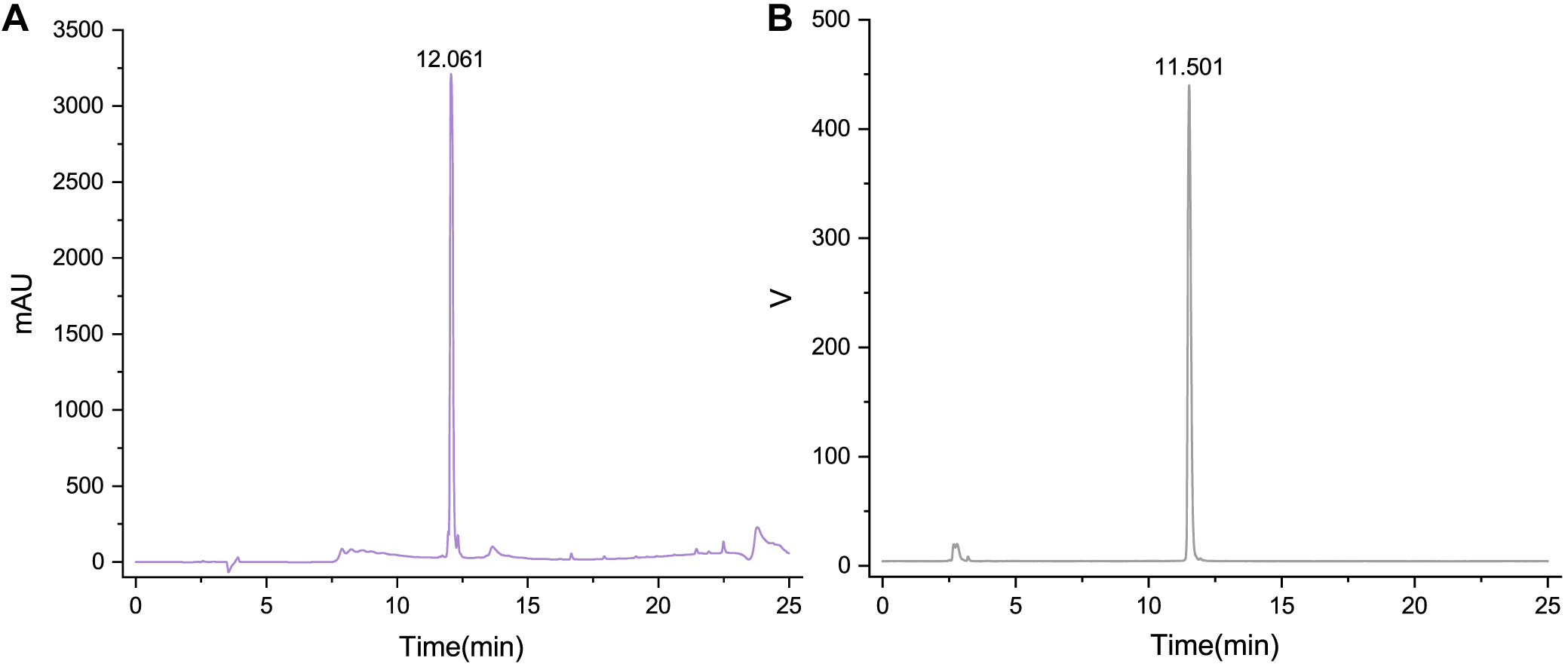
**

Figure S2. HPLC of NOTA-GC-PSMA (A), RT 12.061 min, Area%＞96%. HPLC of ^68^Ga-NOTA-GC-PSMA (B), RT 11.501 min, Area%＞96%.


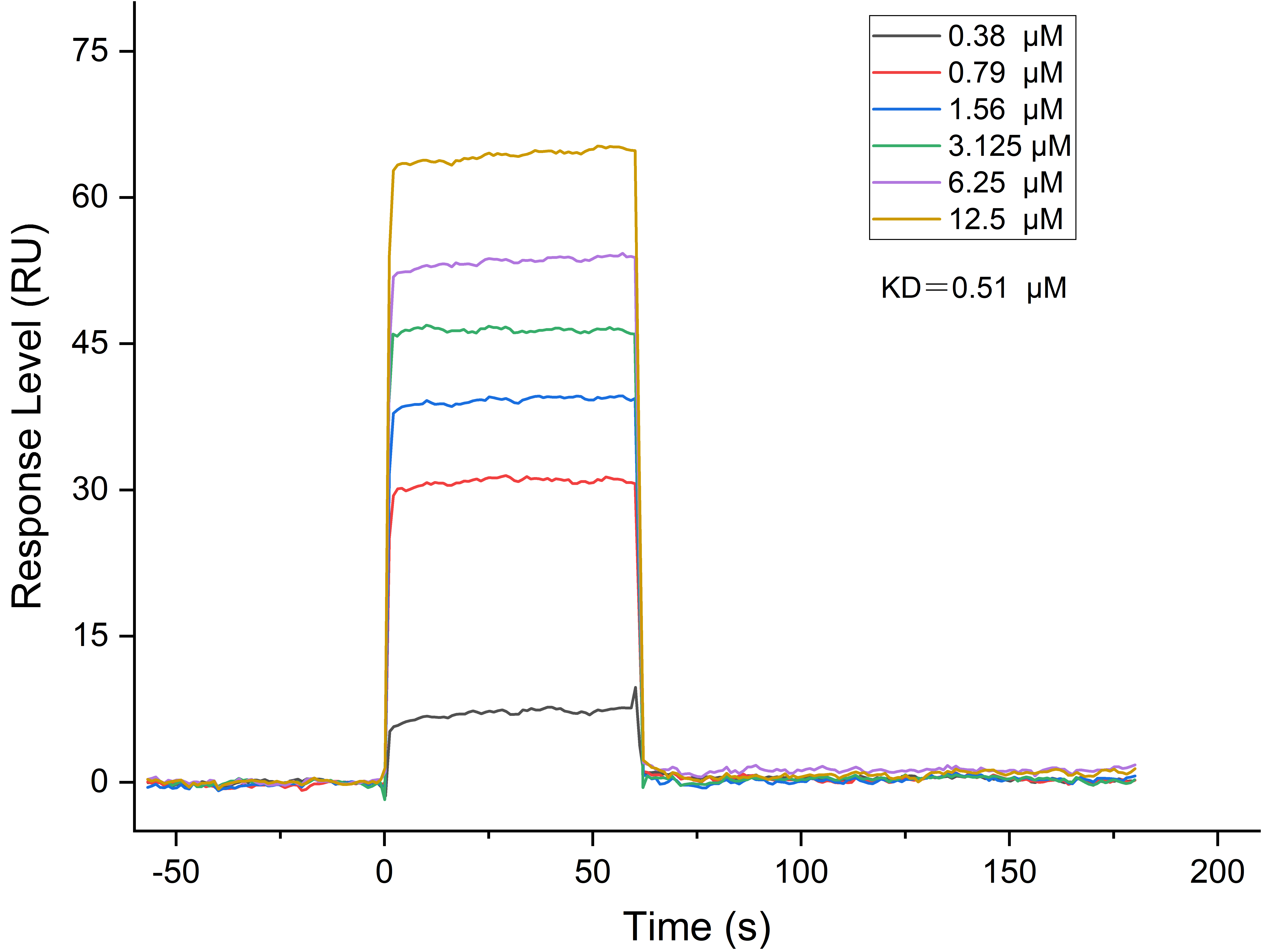


Figure S3. The binding between NOTA-GC-PSMA and PSMA was tested through surface plasmon resonance.


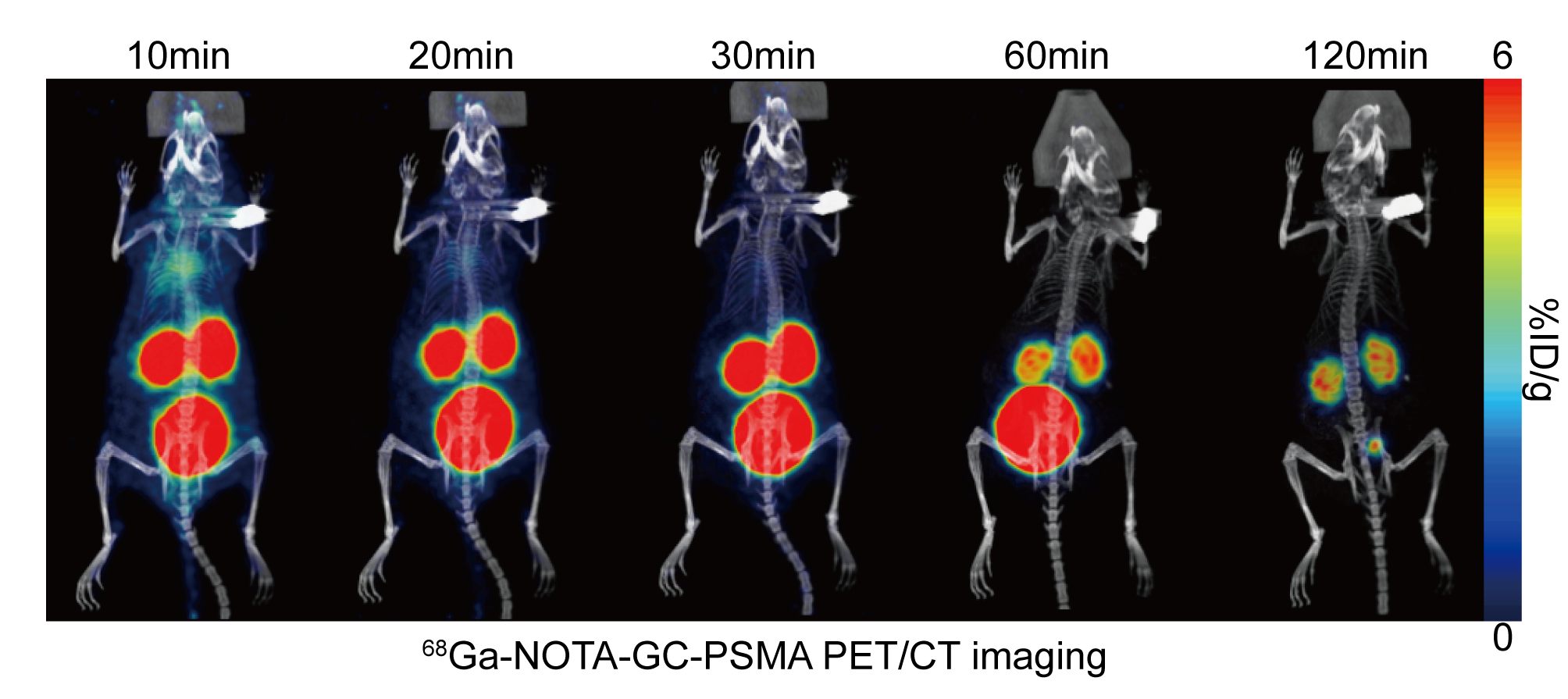


Figure S4. PET/CT imaging of ICR mice (n = 3) with injecting ^68^Ga-NOTA-GC-PSMA.

Table S1. HPLC analysis method of NOTA-GC-PSMA and ^68^Ga-NOTA-GC-PSMA.

|  | Flow rate  (min/mL) | A phase (%)  (0.02% TFA of water) | B phase (%)  (0.04% TFA of acetonitrile) |
| --- | --- | --- | --- |
| 0 min | 1 | 97 | 3 |
| 3 min | 1 | 97 | 3 |
| 20 min | 1 | 0 | 100 |
| 25 min | 1 | 97 | 3 |

Table S2. Cellular experimental data of ^68^Ga-NOTA-GC-PSMA.

| Time(min)  Cell (%10^5^) | 30 | 60 | 90 | 120 |
| --- | --- | --- | --- | --- |
| PC-3 | 0.12 ± 0.01 | 0.11 ± 0.01 | 0.10 ± 0.02 | 0.10 ± 0.01 |
| LNCaP | 1.17 ± 0.16 | 1.35 ± 0.07 | 1.41 ± 0.12 | 1.70 ± 0.13 |
| LNCaP-block | 0.12 ± 0.04 | 0.10 ± 0.00 | 0.13 ± 0.04 | 0.11 ± 0.00 |

Table S3. The pharmacokinetic data of ^68^Ga-NOTA-GC-PSMA was tested by MAS.

| Parameter | Unit | Value |
| --- | --- | --- |
| λ | 1/h | 1.06 |
| T_1/2_ | h | 0.65 |
| T_max_ | h | 0.08 |
| C_max_ | ug/L | 30.36 |
| C_last_ | ug/L | 1.64 |
| AUC_(0-t)_ | h*(ug/L) | 20.14 |
| AUC_(0—inf)__obs | h*(ug/L) | 21.70 |
| Vss_obs | L/kg | 0.74 |

Table S4. The blood sample data of the ^68^Ga-NOTA-GC-PSMA in ICR mice (n = 6).

| Time(min) | Mean ± SD (%ID/g) |
| --- | --- |
| 5 | 5.31 ± 0.81 |
| 15 | 3.15 ± 0.50 |
| 30 | 2.05 ± 0.60 |
| 45 | 1.21 ± 0.39 |
| 60 | 1.10 ± 0.44 |
| 90 | 0.44 ± 0.18 |
| 120 | 0.28 ± 0.14 |
| 180 | 0.27 ± 0.08 |
| 240 | 0.34 ± 0.10 |
| 300 | 0.23 ± 0.14 |

Table S5. Biodistribution of ^68^Ga-NOTA-GC-PSMA in ICR mice (%ID/g, Mean ± SD, n = 5)

| Time(min)  Organ | 10 | 20 | 30 | 60 | 120 |
| --- | --- | --- | --- | --- | --- |
| Brain | 0.89 ± 0.27 | 0.61 ± 0.21 | 0.32 ± 0.10 | 0.12 ± 0.28 | 0.07 ± 0.03 |
| Heart | 2.69 ± 0.32 | 1.72 ± 0.29 | 1.32 ± 0.19 | 0.31 ± 0.13 | 0.15 ± 0.04 |
| Blood | 2.73 ± 0.35 | 1.61 ± 0.18 | 1.44 ± 0.07 | 0.36 ± 0.15 | 0.20 ± 0.03 |
| Liver | 1.84 ± 0.15 | 1.30 ± 0.10 | 0.91 ± 0.10 | 0.34 ± 0.09 | 0.25 ± 0.05 |
| Lung | 1.64 ± 0.16 | 1.06 ± 0.09 | 0.74 ± 0.10 | 0.21 ± 0.09 | 0.10 ± 0.05 |
| Kidney | 12.73 ± 2.22 | 9.79 ± 1.32 | 7.74 ± 1.80 | 4.79 ± 0.13 | 4.63 ± 0.78 |
| Muscle | 0.64 ± 0.20 | 0.48 ± 0.12 | 0.35 ± 0.07 | 0.10 ± 0.06 | 0.05 ± 0.02 |
| Bone | 0.71 ± 0.07 | 0.50 ± 0.06 | 0.39 ± 0.11 | 0.16 ± 0.11 | 0.10 ± 0.05 |

Table S6. The uptake of ^68^Ga-NOTA-GC-PSMA in major organs of LNCaP tumor-bearing mice (%ID/g, Mean ± SD, n = 5)

|  | 10 min | 20 min | 30 min | 60 min | 120 min | Block-60 min |
| --- | --- | --- | --- | --- | --- | --- |
| Brain | 1.0 ± 0.39 | 0.72 ± 0.28 | 0.62 ± 0.09 | 0.25 ± 0.06 | 0.15 ± 0.02 | 0.41 ± 0.26 |
| Heart | 5.93 ± 2.23 | 4.18 ± 1.38 | 3.70 ± 0.81 | 1.43 ± 0.29 | 0.57 ± 0.19 | 1.71 ± 1.54 |
| Liver | 5.24 ± 0.69 | 4.03 ± 0.86 | 2.79 ± 0.79 | 1.69 ± 0.91 | 1.28 ± 1.13 | 2.17 ± 1.22 |
| Lung | 3.24 ± 0.61 | 2.54 ± 0.43 | 2.26 ± 0.23 | 1.02 ± 0.10 | 0.56 ± 0.21 | 1.70 ± 0.96 |
| Kidney | 16.69 ± 3.96 | 13.81 ± 3.49 | 13.17 ± 3.54 | 10.38 ± 2.90 | 8.95 ± 2.63 | 5.07 ± 3.08 |
| Muscle | 1.26 ± 0.49 | 0.87 ± 0.42 | 0.78 ± 0.26 | 0.30 ± 0.18 | 0.09 ± 0.08 | 1.85 ± 3.20 |
| Bone | 0.96 ± 0.32 | 0.68 ± 0.24 | 0.63 ± 0.21 | 0.36 ± 0.34 | 0.11 ± 0.06 | 0.47 ± 0.31 |
| Tumor | 3.01 ± 0.56 | 2.93 ± 0.62 | 3.10 ± 0.20 | 2.28 ± 0.27 | 1.75 ± 0.46 | 1.05 ± 0.58 |
| T/M | 2.65 ± 1.04 | 3.82 ± 1.44 | 5.10 ± 2.27 | 13.87 ± 11.20 | 24.42 ± 20.96 | 4.55 ± 4.27 |
| T/K | 0.18 ± 0.04 | 0.22 ± 0.05 | 0.22 ± 0.08 | 0.20 ± 0.08 | 0.18 ± 0.08 | 0.25 ± 0.11 |
